# Mental Health and Personality Characteristics of University Students at Risk of Smartphone Overdependence

**DOI:** 10.3390/ijerph20032331

**Published:** 2023-01-28

**Authors:** Bo-Kyung Seo, Yoobin Hwang, Hyunseob Cho

**Affiliations:** 1 Addiction Rehabilitation and Social Welfare Department, Eulji University, Uijeongbu 11759, Republic of Korea; 2 School of Psychology, Korea University Graduate School, Seoul 02841, Republic of Korea; 3 Addiction Rehabilitation Counseling Department, Chongshin University, Seoul 06988, Republic of Korea

**Keywords:** college students, mental health, Minnesota multiphasic personality inventory-2-restructured form, MMPI-2-RF, personality, smartphone overdependence

## Abstract

The purpose of this study was to verify the relationship between the risk of smartphone dependence, mental health, and personality traits in university students using the Minnesota Multiphasic Personality Inventory-2-Restructured Form (MMPI-2-RF), and to identify the MMPI-2-RF scales that can predict the risk of smartphone dependence. Of the 772 university students who participated in the study, 163 were in the smartphone overdependence group, accounting for 21.1% of the total survey respondents, which was one in five of those surveyed. High T-scores on the measure indicate greater psychopathology. The smartphone overdependence group had significantly higher T-scores than the general user group on all but three of the MMPI-2-RF scales, and the degree of smartphone overdependence was positively correlated with scores on these scales. There was no difference between the dependent and non-dependent groups on the interpersonal passivity, aesthetic-literary interest, and aggression scales, and scores on these three were not correlated with smartphone dependence. Among the MMPI-2-RF scales, those found to predict the risk of smartphone overdependence were the emotional/internalizing problems, behavioral/externalizing problems, antisocial behavior, cognitive complaints, helplessness/hopelessness, inefficacy, juvenile conduct problems, aggression, interpersonal problems, disconstraint, negative emotionality/neuroticism, and introversion/low positive introversion/low positive emotionality scales. Based on these findings, we propose that effective prevention and intervention for smartphone overdependence must be comprehensive and holistic rather than focusing on specific aspects of mental health or personality. The implications of the findings are discussed.

## 1. Introduction

Smartphones have become ubiquitous and indispensable devices. They are perceived as leisure items and users have positive expectations of smartphones [1]. The rate of smartphone ownership among the Korean population has reached 96.5%, and the household Internet access rate has reached 99.9% [2]. The high prevalence and use of smartphones have led to the development of smartphone overdependence in some. This is a problem that has emerged along with the expansion of the Internet supply network in Korea, the introduction of policies to resolve the digital divide, and the rapid development of electronic communication devices. The 2021 Smartphone Overdependence Survey found smartphone overdependence to affect 24.2% of the entire population, which was a 0.9% increase from 2020. By age group, adolescents have the highest rate of overdependency at 37.0%, followed by children at 28.4%, adults at 23.3%, and older adults aged 60+ at 17.5%, with increases apparent across all age groups [1]. By the decade of life, the rates of smartphone overdependence were found to be 37.0% among teenagers, 31.5% among those in their 20s, 23.8% for those in their 30s, 20.1% for those in their 40s, 19.2% for those in their 50s, and 17.5% for those in their 60s. It can be seen that the rate of overdependence decreases with increasing age.

The annual national statistics on smartphone overdependence rates show the highest rate of smartphone overdependence in teenagers, followed by those in their 20s, with college students accounting for 33.1% of those with smartphone overdependence in 2022, an increase of 0.9% compared to 2021 [1]. Around 3 out of 10 college students are smartphone overdependent. Studies on smartphone overdependence in college students have investigated a variety of risk factors, including college life adaptation, stress, anxiety, self-esteem, family communication, attachment, and parenting style [3,4,5]. According to research results, they suggested intervention programs to address smartphone overdependence, depression, stress, and anxiety, and to boost the ego and sense of self-efficacy in college students with smartphone overdependence [6,7,8,9,10]. While there have been previous studies into the relationships between mental health and psychological characteristics [8,11,12], few studies have comprehensively analyzed the mental health and personality characteristics of college students with smartphone overdependence. Therefore, this study aimed to identify the relationships between smartphone overdependence, mental health, and personality traits of college students using the Minnesota Multiphasic Personality Inventory-2-Reconstructed Form (MMPI-2-RF), the validity and reliability of which are well-established [13]. It also aimed to determine whether any of the MMPI-2-RF scales could be used to predict the risk of smartphone dependence among this population.

### 1.1. Smartphone Overdependence

Griffths (2005) defined behavioral addiction as any behavior that fulfills the six components of addiction, that is, salience, mood modification, tolerance, withdrawal, conflict, and relapse [14]. However, Billieux et al. (2015) reported the lack of empirical evidence and studies supporting mobile phone use as a behavioral addiction [15]. They suggested that longitudinal and experimental research is needed to obtain behavioral and neurobiological correlates of problematic mobile phone use. They suggested a pathway model of problematic mobile phone use that allows us to understand the etiology and course. The suggested three pathways leading to problematic mobile phone use are an excessive reassurance pathway, an impulsive-antisocial pathway, and an extraversion pathway.

Unlike the Billieux’s pathway model of problematic mobile phone use, smartphone overdependence focuses on the symptom and consequence of the mobile phone use. In those with smartphone overdependence, use becomes excessive and the user’s ability to control it decreases [16]. Aspects of smartphone overdependence include salience, control failure, and serious consequences. Salience refers to the increasing importance and prominence of the smartphone until it becomes the most important element of the individual’s life. Self-control failure refers to the inability to exercise autonomous control over smartphone use. Serious consequences refer to continued excessive smartphone use despite negative physical, psychological, and social consequences [17].

Although the researchers showed evidence about the behavioral addiction, there are still debates about the existence of behavioral addiction [18,19]. The Ministry of Science and Information and Communications Technology (ICT) and the National Information Society Agency in Korea are responsible for the prevention of adverse effects of ICT and intelligent information services in accordance with the 2020 Framework Act on Intelligent Informatization. The Ministry of Science and ICT changed the term from smartphone addiction to smartphone overdependence, and the new term was approved by the National Statistical Office in 2016 and is used in nationally approved statistics. This change in terminology represents a change in the government’s perspective on smartphone use. The existing smartphone addiction prevention and intervention policies were based on a pathological point of view on smartphone use. The new term recognizes that high levels of smartphone use are now very normal, and that there is also a “normal” level of dependence on these devices. “Overdependence” distinguishes pathological use by its amount and its effects on daily life [16].

In the 11th revision of the International Classification of Diseases (ICD-11), the World Health Organization officially recognized pathological levels of video game use through its inclusion of gaming disorder as an official disease [20]. However, while gaming disorder may involve excessive use of internet games on smartphones, smartphone overuse itself is not officially recognized as a disorder. Nevertheless, the concept of smartphone overdependence in Korea is based on the same distinction between normal behaviors and psychological pathologies used by the ICD-11—interference with daily life. This is used to define smartphone overdependence and by the ICD-11 and other sources of diagnostic criteria to define addictive behaviors with no biological component, as well as distinguishing between, for example, normal worrying and anxiety disorders, or normal fears and phobias.

### 1.2. Smartphone Overdependence and Mental Health

Smartphone overdependence in college students has shown correlations with depression, anxiety, impulsivity, low self-efficacy, and poor interpersonal relationships. College students with high levels of depression or anxiety tend to fall into smartphone overdependence to relieve negative emotions, and, as a result, have difficulty adjusting to college life [10,21,22,23]. Depression and impulsiveness significantly increase the risk of smartphone overdependence, and this can affect interpersonal relationships through conflicts with friends or fewer face-to-face conversations [23].

Impulsivity is the tendency to act for immediate gratification without consideration of consequences. People with high impulsivity tend to seek novel, immediate, sensual, and diverse stimuli and have a high risk of smartphone overdependence [8]. College students with low self-efficacy have shown a higher tendency to smartphone overdependency than those with high self-efficacy [21,24]. Smartphone overdependence has also been found to relate to interpersonal competence [25,26].

Most studies on smartphone overdependence in college students have focused on the relationship between smartphone overdependence and mental health. However, to effectively identify those at risk, the relevant personality characteristics must also be established.

### 1.3. Smartphone Overdependence and Multiphasic Personality Inventory (MMPI-II-RF)

In the present study, we evaluated mental health and personality characteristics using the MMPI-2-RF [27], a restructured version of the Minnesota Multiphasic Personality Inventory-2 (MMPI-2) [28]. This is a test developed based on pathological theory [27] created empirically by building on the high inter-scale correlations of the MMPI-2. The MMPI-2-RF has excellent discriminatory power between scales, having increased the independence of each of its scales by strengthening response sincerity by reducing the number of items and thereby lowering response fatigue. The MMPI-2-RF has a three-layer hierarchical structure, with higher-order scales, reconstructed clinical scales, and specific problem scales. It systematically and comprehensively identifies mental health and personality characteristics and reflects psychopathology and modern personality models and frameworks [29].

Studies that have used the MMPI-2-RF to identify the relationship between smartphone overdependence and mental health and personality traits are very limited. Lim (2005) analyzed the relationship between internet addiction in college students and trait measures on the MMPI-2 [30]. They found that the internet addiction group had significantly higher scores than the general user group on all clinical scales of the MMPI-2, except the masculinity-femininity (MF) scale. Significantly higher scores were seen on the depression, antisocial, paranoid, obsessive-compulsive, and introversion scales. Other studies of adults and college students found those with internet addiction showed significantly higher levels of hypochondria, depression, antisociality, obsessive-compulsiveness, schizotypal qualities, and introversion [31,32]. Jung (2016) found significantly higher scores on the hypochondria, paranoia, obsessive-compulsive, and schizophrenia scales in a smartphone addiction group than in a general user group among college students [32]. A significant correlation was found between internet addiction and scores on the obsessive-compulsive and introversion scales, but only the obsessive-compulsive scale was correlated with smartphone addiction [32].

In a study that analyzed the personality and mental health of internet-addicted adolescents using the MMPI-A (Minnesota Multiphasic Personality Inventory-Adolescents, 1992), they found higher scores among internet addicts on the depression, antisociality, obsessive-compulsive, schizophrenia, and introversion scales [33,34,35]. An Italian study using the MMPI-A found adolescents with problematic internet use had higher scores on the paranoia and schizophrenia scales. This is coded as type 6-8/8-6 and describes adolescents with immature egos that manifest as unregulated emotions and behaviors and a reduced sense of reality [36].

Although the MMPI is one of the most used diagnostic tools in the counseling research, there have been no previous studies that have used the MMPI to predict the risk of smartphone overdependence.

Therefore, this study aimed to identify the relationships between smartphone addiction, mental health, and personality characteristics among college students using the MMPI-2-RF. In addition, we outline the personality profile of those at risk of smartphone overdependence and suggest interventions that can be applied in counseling. The research questions were as follows:

1. How do scores on MMPI-2-RF scales differ between a smartphone overdependence group and a general user group?
2. What are the relationships between smartphone overdependence and scores on the MMPI-2-RF scales?
3. Which of the MMPI-2-RF scales can predict smartphone overdependence?

## 2. Materials and Methods

### 2.1. Participants

The participants in this study were 823 sophomores at a university in Gyeonggi-do, Korea. The mean age of the participants was 20.3 ± 0.88 years. There were 286 (37%) male participants and there were 486 (63%) female participants. The data were collected by the counseling center of the university, which surveyed the mental health of the students in a year. The participants were informed that the collected data could be used only for the statistics and for the research. They read the information about the survey and signed that they agreed the participation in the survey.

Based on the scores recommended in the MMPI-2-RF manual (i.e., Cannot Say (CNS-r) > 18, Variable Response Inconsistency (VRIN-r) > 79 T, True Response Inconsistency (TRIN-r) > 79 T, Infrequent Responses (F-r) > 119 T, Infrequent Psychopathology Responses (Fp-r) > 99 T, Uncommon Virtues (L-r) > 79 T, or Adjustment Validity (K-r) > 69 T), 51 people were excluded [30]. After these exclusions, 772 respondents were included in our analysis.

### 2.2. Instruments

#### 2.2.1. Smartphone Overdependence Scale

The Korea National Information Society Agency (2016) has developed a Smartphone Overdependence Scale, which incorporates measures of three sub-elements of smartphone overdependence: salience, self-control failure, and serious consequences [16]. Salience refers to the importance of smartphone use to the respondent. Self-control failure refers to the extent to which the respondent finds it difficult to control their smartphone use. Serious consequences refer to conflicts the respondent has experienced with those around them, physical discomfort, and problems in their home, school, and work life due to their excessive smartphone use.

The Smartphone Overdependence Scale comprises 10 statements, to which responses are given on a four-point Likert scale ranging from “not at all” (1 point) to “very much so” (4 points). Possible scores range from 10 to 40. Those with scores of 10–23 points are classified as general smartphone users; those scoring 24–28 points are users with a potential risk of overdependence; those scoring 29–40 points are classed as high-risk overdependence users. The annual rate of smartphone overdependence is a nationally approved statistic (No. 120019) announced by the Ministry of Science and ICT every year. The rates given are the sum of those administered the Smartphone Overdependence Scale whose scores indicate potential risk and high risk as a percentage of the total number of participants [1]. We utilized this scale in the same way in the present study, with the potential risk and high-risk scoring participants combined into a smartphone overdependence group. We found the internal consistency of this test to have a high Cronbach’s α of 0.889.

#### 2.2.2. Minnesota Multiphasic Personality Inventory-2 Restructured Form (MMPI-2-RF)

The MMPI-2-RF (2008, 2011) has a total of 338 items, each of which can be answered with “yes” or “no” [27]. The MMPI-2-RF incorporates 50 scales and is organized in a hierarchical structure. It is composed of 8 validity scales, 3 high-order scales, 9 reconstructive clinical scales, 23 specific problem scales (5 somatic/cognitive symptom scales, 9 internalization scales, 4 externalization scales, and 5 interpersonal relationship scales), 2 interest scales, and 5 scales of personality psychopathology [27].

MMPI-2-RF is used in a variety of areas, such as criminal courts, child custody issues, selection of public safety personnel, recruitment of law enforcement officers or prosecutors, evaluation of military service accessibility, psychological evaluation before employment, and evaluation of psychological factors that affect compliance, with various treatments and results [13,37]. A paper-and-pencil test was conducted and scored by a computer program. The subscales have 5 items to 41 items. The manual explains that the scales with few items have a low Cronbach’s alpha, and the scales with many items have a high Cronbach’s alpha. The Cronbach’s alpha of scales ranges from 0.41 of the HLP ~ 0.79 of the THD in Korean version of MMPI-2-RF. We found the internal consistency of this test to have a very high Cronbach’s α of 0.918.

### 2.3. Statistical Analysis

Data in this study were analyzed using SPSS 22.0 (IBM Corp., Armonk, NY, USA). Means, standard deviations, and percentages of demographic characteristics and key variables were calculated.

To identify the MMPI-2-RF scales for which there was a significant difference between the scores of the smartphone overdependence group and the general user group, a *t*-test was conducted with the two groups as independent variables and the higher-order scales, reconstructed clinical scales, specific problem scales, interest scales, and personality pathology five-factor scales as dependent variables.

When scoring the MMPI-2-RF, T-scores for each scale are calculated from the raw scores; therefore, T-scores were also used for scale scoring in this study. For comparisons between the two groups, χ^2^ tests, *t*-tests, and multivariate ANOVA were performed. Pearson’s correlation coefficient was used to analyze the relationships between variables.

To identify the MMPI-2-RF scales that predict the risk of smartphone overdependence, logistic regression analysis was performed using the MMPI-2-RF scale as an independent variable and the Smartphone Overdependence Scale as a dependent variable. The MMPI-2-RF scales are hierarchically linked. This means that, when scores for these scales are analyzed together, multicollinearity may occur. To avoid this, logistic regression analysis was performed separately for each of the higher-dimensional, reconstructed clinical-specific problem scales and personality psychopathology scales. The significance level for all analyses performed was set at *p* < 0.05.

## 3. Results

### 3.1. Rate of Smartphone Overdependence

Among the 772 college students who participated in the study, 163 were in the smartphone overdependence group, accounting for 21.1%, or one in five, of the total sample. There was a significant difference in the male-to-female ratio between the smartphone overdependence group and the general user group (χ^2^ = 7.894, *p* < 0.01). Among male participants, the smartphone overdependency rate was 15.7%; among female participants, it was 24.3% (Table 1). The mean scores on the Smartphone Overdependence Scale were 27.35 (SD = 3.10) in the smartphone overdependence group and 17.33 (SD = 3.96) in the general user group, which was a significant difference. Those in the smartphone overdependence group also had significantly higher scores than the general user group in the sub-factors of control failure, salience, and serious consequences (Table 1).

### 3.2. Mental Health and Personality Characteristics of Smartphone Overdependence Group

**Figure 1:**
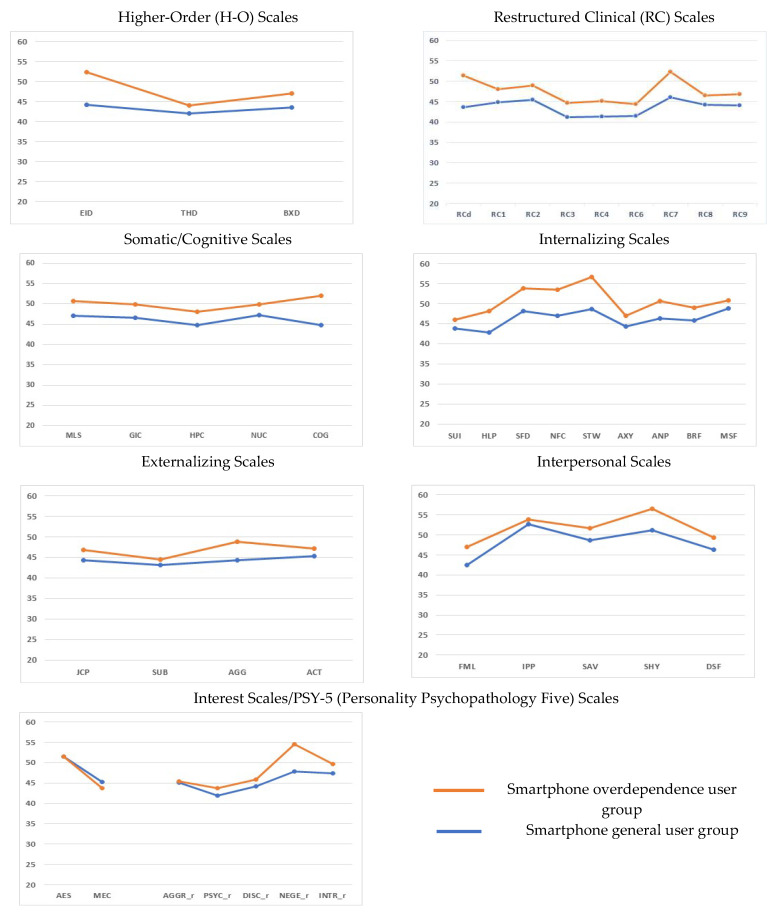
Comparison of the mean scores of the smartphone overdependence group and the general user group on each Minnesota Multiphasic Personality Inventory-2-Revised Form scale.

As shown in Table 2, the smartphone overdependence group had significantly higher scores than the general user group on all but three of the MMPI-2-RF scales. The three scales with no significant between-group differences were interpersonal passivity (IPP), aesthetic-literary interest (AES), and aggression (AGGR_r) (Figure 1).

### 3.3. Correlation between Smartphone Overdependence and MMPI-2-RF Scale Scores

As shown in Table 3, Scores on the Smartphone Overdependence Scale were significantly correlated with all but three of the MMPI-2-RF scales. These were the same scales with no significant between-group differences, the IPP, AES, and AGGR_r scales. All but one of the significant correlations were positive, with only the mechanical-physical interest scale (MEC) scores showing a negative correlation with Smartphone Overdependence Scale scores (r = −0.134, *p* < 0.001). Thus, the degree of smartphone overdependence was positively correlated with emotional, cognitive, and behavioral problems, interpersonal issues, and personality pathologies.

### 3.4. Association of MMPI-2-RF Scales with Smartphone Overdependence

Separate binary logistic regression analysis was performed to identify the predictive scales of the MMPI-2-RF for the smartphone overdependence.

The following scales were significant predictors of smartphone overdependence: the emotional/internalizing problem (EID: OR = 1.051, *p* = 0.000), behavioral/externalizing problem (BXD: OR = 1.059, *p* = 0.000), the antisocial behavior (RC4: OR = 1.050, *p* = 0.002), the cognitive complaint (COG) (COG: OR = 1.064, *p* = 0.000), the helplessness/hopelessness (HLP: OR = 1.323, *p* = 0.002), the inefficacy (NFC: OR = 1.044, *p* = 0.001), juvenile conduct problems (JCP: OR = 1.036, *p* = 0.008), aggression (AGG_r: OR = 1.055, *p* = 0.000), the family problems (FML: OR = 1.043, *p* = 0.000) and shyness (SHY: OR = 1.031, *p* = 0.001), disconstraint-revised (DISC_r: OR = 1.034, *p* = 0.016), negative emotionality/neuroticism-revised (NEGE_r: OR = 1.046, *p* = 0.000), and introversion/low positive emotionality-revised (INTR_r: OR = 1.030, *p* = 0.007) (Table 4).

## 4. Discussion

The purpose of this study was to identify the relationships between smartphone dependence, mental health, and personality traits in university students using the MMPI-2-RF, and to identify the MMPI-2-RF scales that can predict the risk of smartphone dependence.

Of the 772 university students who participated in this study, 163 were classed as smartphone overdependent based on their scores on the Smartphone Overdependence Scale. This represented 21.1%, or one in five, of the sample. The proportion of female participants with smartphone overdependence was 24.3%, which was 8.6 percentage points higher than the proportion of males (15.7%). This is consistent with the results of previous studies [38,39]. Sex differences may be related to the usage characteristics of smartphones. In a study by Lee (2015) of activities for which smartphones were used, men used them most frequently to watch videos, followed by accessing social network services (SNS), and listening to music. Women most often used their smartphones for instant messaging, followed by SNS, and listening to music. She found that the greater the use of the smartphone for communication purposes, the higher the degree of addiction [39]. Cho and Jeon (2016) confirmed that gender is significantly related to smartphone addiction, with women having a 3.94 times higher rate of smartphone overdependence than men [38].

In the present study, the smartphone overdependence group had significantly higher T-scores than the general user group on all but three of the MMPI-2-RF scales. These three were the IPP, AES, and AGGR_r scales. Thus, the smartphone overdependence group scored higher than the general user group in the higher-order, reconstructed clinical, specific problem (somatic/cognitive, internalizing, externalizing, and interpersonal scales), and personality pathology five scales.

Lim (2005) reported that college students at risk of internet addiction showed higher scores on all clinical scales but the femininity (MF) scale than general users using the MMPI-2 and MMPI-A [30]. Jung (2016) found that university students with smartphone overdependence differed from general users on the health concerns, paranoia, obsessive-compulsive, and schizophrenia scales [32]. These differing results appear to be attributable to differences in participant characteristics and the smartphone addiction scales used. Further repetition of research is needed to verify the personality profile of individuals with smartphone addiction.

We found significant positive correlations between the degree of smartphone overdependence and all MMPI-2-RF scales except the IPP, AES, AGGR_r, and MEC scales, with the latter having a significant negative correlation. Thus, the degree of smartphone dependence is positively correlated with emotional, cognitive, behavioral, and interpersonal problems, and with pathological personality characteristics. Other studies have also reported relationships between smartphone overdependence and psychological factors such as depression, anxiety, impulsivity, and interpersonal issues [10,21,26,40].

Our findings suggest that those at risk of smartphone overdependence may suffer from a range of emotional, behavioral, cognitive, and interpersonal issues. Interventions that target only the surface problems in smartphone overdependence, such as usage time, content, habits, academic performance and attitudes, or tardiness and absenteeism are likely to be ineffectual. The core issues, including negative emotions, problematic behaviors, irrational thinking, low self-efficacy, interpersonal problems, and personality constraints, must also be addressed.

The MMPI-2-RF scales that we found to be predictors of smartphone overdependence risk were the EID and BXD higher-order scales; the RC4 reconstructed clinical scale; the COG somatic/cognitive scale; the HLP and NFC internalizing scales; the JCP and AGG externalizing scales; the FML and SHY interpersonal scales; and the disconstraint (DISC_r), negative emotionality/neuroticism (NEGE_r), and the introversion/low positive emotionality (INTR_r) PSY-5 scales.

The results indicate that the likelihood that university students with the high scores on these scales belong to the smartphone overdependence group is higher than the likelihood belonging to the smartphone general user group. The personality characteristic of the smartphone overdependence group we found could be described as follows. Firstly, they have high negative emotionality. Therefore, they are depressive, shy, pessimistic, and emotionally unstable. Regarding this emotional characteristic, they could have low self-esteem, low self-confidence, and interpersonal problems, including family problems. Secondly, they have aggressive internalizing or externalizing behaviors. They could have suicidal thoughts, and they could be self-aggressive or aggressive verbally or behaviorally towards others. Regarding aggression of the smartphone addictive users, Nuri et al. (2021) reported that smartphone addiction predicts hospitality, physical aggression, verbal aggression, and anger among university students in Cyprus [41]. Kim et al. (2015) demonstrated an association between smartphone addiction and aggression in Korea [42]. Yilmaz et al. (2023) reported that aggression was a predictor of smartphone addiction [43].

These scales can be utilized to predict smartphone overdependence among university students.

## 5. Conclusions

In this study, we used the MMPI-2-RF to identify the relationships between smartphone overdependence and the mental health and personality characteristics of university students. We also identified those MMPI-2-RF scales that can predict smartphone overdependence risk among university students. We propose that effective prevention and intervention for smartphone overdependence must be comprehensive and holistic rather than focusing on specific aspects of mental health or personality.

This study had some limitations. First, the sample used was university students in Gyeonggi-do, Korea, and, therefore, does not represent all university students. To generalize our results, they must be verified with university students nationwide. Second, no comorbidity diagnoses or information was gathered from participants. If common comorbidities of smartphone dependence were identified, we would have a clearer picture of the relationships between smartphone dependence risk, mental health, and personality traits. Third, this study used the entire MMPI-2-RF. However, increased efficiency and economy and more precise results could be achieved through intensive research using specific MMPI-2-RF scales.

Mental health and personality traits contribute to vulnerability, resilience, and the ability to adapt well to change. They can affect the pattern and prognosis of mental disorders. This study analyzed the relationship between smartphone overdependence and mental health and personality characteristics among a generation that lives with the ongoing accelerated development of diverse digital devices.

## Figures and Tables

**Table 1:** A comparison of measurements of the aspects of smartphone overdependence (Smart Rest Center, 2022) in the smartphone overdependence group and the general user group.

		Smartphone Overdependence User Group (N = 163)	Smartphone General User Group (N = 609)	Total (N = 772)	χ^2^/*t*
sex	male	45 (15.7)	241 (84.3)	286 (37.0)	7.894 **
	female	118 (24.3)	368 (75.7)	486 (63.0)	
smartphone overdependence		25.83 (2.04)	18.61 (3.51)	20.14 (4.40)	25.15 ***
	control failure	9.19 (1.07)	6.51 (1.70)	7.08 (1.93)	19.08 ***
	salience	8.23 (1.19)	5.89 (1.40)	6.38 (1.66)	19.59 ***
	problematic results	8.41 (1.15)	6.21 (1.61)	6.67 (1.82)	15.76 ***

** *p* < 0.01, *** *p* < 0.001.

**Table 2:** Comparison of the mean MMPI-2-RF scale scores of the smartphone overdependence group and the general user group.

	MMPI-2-RF Scales	Smartphone Overdependence User Group	Smartphone General User Group	*t*
		(N = 163)	(N = 609)	
		M (SD)	M (SD)	
Higher-Order (H-O) Scales	EID	52.28 (13.03)	44.14 (10.32)	71.12 ***
	THD	44.01 (6.76)	42.02 (5.41)	15.57 ***
	BXD	47.01 (7.35)	43.55 (6.45)	34.97 ***
Restructured Clinical (RC) Scales	RCd	51.40 (11.75)	43.69 (9.81)	72.91 ***
	RC1	48.00 (9.39)	44.80 (7.40)	21.28 ***
	RC2	48.99 (8.91)	45.39 (8.13)	24.21 ***
	RC3	44.63 (7.34)	41.21 (6.75)	31.94 ***
	RC4	45.17 (7.09)	41.32 (6.33)	45.24 ***
	RC6	44.39 (7.30)	41.55 (6.00)	26.18 ***
	RC7	52.33 (10.40)	46.03 (8.13)	68.07 ***
	RC8	46.55 (8.45)	44.17 (6.40)	15.43 ***
	RC9	46.82 (7.63)	44.12 (7.12)	17.94 ***
Specific Problems				
Somatic/Cognitive Scales	MLS	50.76 (9.46)	47.05 (8.38)	23.89 ***
	GIC	49.89 (10.94)	46.64 (8.81)	15.70 ***
	HPC	48.02 (10.72)	44.72 (8.04)	18.58 ***
	NUC	49.82 (8.38)	47.33 (7.07)	14.66 ***
	COG	52.02 (11.95)	44.78 (8.39)	78.80 ***
Internalizing Scales	SUI	45.93 (7.11)	43.85 (5.46)	16.22 ***
	HLP	48.10 (10.30)	42.77 (7.06)	59.25 ***
	SFD	53.88 (11.58)	48.21 (10.47)	36.09 ***
	NFC	53.40 (10.14)	46.92 (8.42)	69.65 ***
	STW	56.64 (12.65)	48.72 (10.81)	35.78 ***
	AXY	47.05 (7.43)	44.32 (5.66)	25.93 ***
	ANP	50.58 (11.27)	46.30 (9.60)	23.71 ***
	BRF	49.01 (8.60)	45.82 (7.25)	22.94 ***
	MSF	50.77 (11.24)	48.88 (10.03)	4.33 *
Externalizing Scales	JCP	46.78 (7.22)	44.37 (6.62)	16.36 ***
	SUB	44.42 (6.78)	43.09 (5.91)	6.13 *
	AGG	48.77 (9.52)	44.32 (7.48)	40.29 ***
	ACT	47.06 (8.05)	45.36 (8.06)	5.75 *
Interpersonal Scales	FML	47.05 (10.60)	42.54 (7.91)	35.73 ***
	IPP	53.81 (11.24)	52.73 (10.62)	1.30
	SAV	51.63 (12.93)	48.59 (11.06)	9.01 **
	SHY	56.56 (12.68)	51.21 (10.98)	28.56 ***
	DSF	49.26 (10.37)	46.39 (9.26)	11.75 ***
Interest Scales	AES	51.42 (9.35)	51.47 (9.58)	0.004
	MEC	43.69 (7.65)	45.27 (8.60)	4.54 *
Personality Psychopathology Five (PSY-5) Scales, Revised	AGGR_r	45.45 (9.03)	45.09 (7.64)	0.26
	PSYC_r	43.69 (7.09)	41.91 (5.97)	10.53 ***
	DISC_r	45.93 (7.12)	44.15 (7.27)	7.75 **
	NEGE_r	54.59 (12.14)	47.88 (10.39)	49.83 ***
	INTR_r	49.66 (9.37)	47.34 (9.26)	8.06 **

EID: emotional/internalizing dysfunction, THD: thought dysfunction, BXD: behavioral/externalizing dysfunction, RCd-(dem): demoralization, RC1-(som): somatic complaints, RC2-(lpe): low positive emotions, RC3-(cyn): cynicism, RC4-(asb): antisocial behavior, RC6-(per): ideas of persecution, RC7-(dne): dysfunctional negative emotions, RC8-(abx): aberrant experiences, RC9-(hpm): hypomanic activation, MLS: malaise, GIC: gastro-intestinal complaints, HPC: head pain complaints, NUC: neurological complaints, COG: cognitive complaints, SUI: suicidal/death ideation, HLP: helplessness/hopelessness, SFD: self-doubt, NFC: inefficacy, STW: stress/worry, AXY: anxiety, ANP: anger proneness, BRF: behavior-restricting fears, MSF: multiple specific fears, JCP: juvenile conduct problems, SUB: substance abuse, AGG: aggression, ACT: activation, FML: family problems, IPP: interpersonal passivity, SAV: social avoidance, SHY: shyness, DSF: disaffiliativeness, AES: aesthetic-literary interests, MEC: mechanical-physical interests, AGGR-r: aggressiveness-revised, PSYC-r: psychoticism-revised, DISC-r: disconstraint-revised, NEGE-r: negative emotionality/neuroticism-revised, INTR-r: introversion/low positive emotionality-revised; * *p* < 0.05, ** *p* < 0.01, *** *p* < 0.001.

**Table 3:** Correlation between Smartphone Overdependence Scale Scores and MMPI-2-RF Scale Scores.

MMPI-2-RF Scales		r
Higher-Order (H-O) Scales	EID	0.333 ***
	THD	0.154 ***
	BXD	0.252 ***
Restructured Clinical (RC) Scales	RCd	0.357 ***
	RC1	0.192 ***
	RC2	0.176 ***
	RC3	0.219 ***
	RC4	0.246 ***
	RC6	0.178 ***
	RC7	0.351 ***
	RC8	0.185 ***
	RC9	0.242 ***
Specific Problems		
Somatic/Cognitive Scales	MLS	0.195 ***
	GIC	0.135 ***
	HPC	0.160 ***
	NUC	0.132 ***
	COG	0.332 ***
Internalizing Scales	SUI	0.158 ***
	HLP	0.237 ***
	SFD	0.232 ***
	NFC	0.356 ***
	STW	0.275 ***
	AXY	0.176 ***
	ANP	0.277 ***
	BRF	0.223 ***
	MSF	0.107 **
Externalizing Scales	JCP	0.123 ***
	SUB	0.107 **
	AGG	0.265 ***
	ACT	0.162 ***
Interpersonal Scales	FML	0.225 ***
	IPP	0.019
	SAV	0.094 **
	SHY	0.223 ***
	DSF	0.109 **
Interest Scales	AES	−0.043
	MEC	−0.134 ***
PSY-5 (Personality Psychopathology Five) Scales, Revised	AGGR_r	0.031
	PSYC_r	0.150 ***
	DISC_r	0.112 **
	NEGE_r	0.330 ***
	INTR_r	0.087 *

EID: emotional/internalizing dysfunction, THD: thought dysfunction, BXD: behavioral/externalizing dysfunction, RCd-(dem): demoralization, RC1-(som): somatic complaints, RC2-(lpe): low positive emotions, RC3-(cyn): cynicism, RC4-(asb): antisocial behavior, RC6-(per): ideas of persecution, RC7-(dne): dysfunctional negative emotions, RC8-(abx): aberrant experiences, RC9-(hpm): hypomanic activation, MLS: malaise, GIC: gastro-intestinal complaints, HPC: head pain complaints, NUC: neurological complaints, COG: cognitive complaints, SUI: suicidal/death ideation, HLP: helplessness/hopelessness, SFD: self-doubt, NFC: inefficacy, STW: stress/worry, AXY: anxiety, ANP: anger proneness, BRF: behavior-restricting fears, MSF: multiple specific fears, JCP: juvenile conduct problems, SUB: substance abuse, AGG: aggression, ACT: activation, FML: family problems, IPP: interpersonal passivity, SAV: social avoidance, SHY: shyness, DSF: disaffiliativeness, AES: aesthetic-literary interests, MEC: mechanical-physical interests, AGGR-r: aggressiveness-revised, PSYC-r: psychoticism-revised, DISC-r: disconstraint-revised, NEGE-r: negative emotionality/neuroticism-revised, INTR-r: introversion/low positive emotionality-revised; * *p* < 0.05, ** *p* < 0.01, *** *p* < 0.001.

**Table 4:** Association between smartphone overdependence and MMPI-2-RF scales assessed by logistic regression analysis.

	OR (95% CI)	*p*
Higher-Order (H-O) Scales		
EID	1.051 (1.034–1.069)	0.000
BXD	1.059 (1.029–1.090)	0.000
Restructured Clinical (RC) Scales		
RC4	1.050 (1.018–1.084)	0.002
Somatic/Cognitive Scales		
COG	1.064 (1.042–1.085)	0.000
Internalizing Scales		
HLP	1.323 (0.871–2.009)	0.002
NFC	1.044 (1.018–1.071)	0.001
Externalizing Scales		
JCP	1.036 (1.009–1.063)	0.008
AGG	1.055 (1.030–1.080)	0.000
Interpersonal Scales		
FML	1.043 (1.022–1.064)	0.000
SHY	1.031 (1.013–1.049)	0.001
PSY-5 (Personality Psychopathology Five) Scales, Revised		
DISC_r	1.034 (1.006–1.063)	0.016
NEGE_r	1.046 (1.027–1.064)	0.000
INTR_r	1.030 (1.008–1.052)	0.007

EID, emotional/internalizing dysfunction; BXD, behavioral/externalizing dysfunction; CI, confidence interval; RC4, antisocial behavior; COG, cognitive complaints; HLP, helplessness/hopelessness; NFC, inefficacy; JCP, juvenile conduct problems; AGG, aggression; FML, family problems; SHY, shyness; DISC_R, disconstraint-revised; NEGE_R, negative emotionality/neuroticism-revised; INTR_R, introversion/low positive emotionality-revised. The reference group was the smartphone overdependence group.

## Data Availability

The data presented in this study are available on request from the corresponding author. The data are not publicly available due to privacy.
